# A national survey of ambient air pollution health literacy among adult residents of Taiwan

**DOI:** 10.1186/s12889-021-11658-z

**Published:** 2021-08-31

**Authors:** Wen-Hsuan Hou, Yi-Chin Huang, Chien-Yeh Lu, I-Chen Chen, Pei-Chen Lee, Ming-Yeng Lin, Yu-Chen Wang, Lilis Sulistyorini, Chung-Yi Li

**Affiliations:** 1 School of Gerontology Health Management & Master Program in Long-Term Care, College of Nursing, Taipei Medical University, Taipei, Taiwan; 2 Department of Physical Medicine and Rehabilitation, Department of Geriatric Medicine, & Department of Education, Taipei Medical University Hospital, Taipei, Taiwan; 3 Graduate Institute of Clinical Medicine, College of Medicine, Taipei Medical University, Taipei, Taiwan; 4 Department of Public Health, College of Medicine, National Cheng Kung University, #1, University Rd, Tainan, Taiwan 701; 5 Health Promotion Administration, Ministry of Health and Welfare, Taipei, Taiwan; 6 Department of Health Care Management, National Taipei University of Nursing and Health Sciences, Taipei, Taiwan; 7 Department of Environmental and Occupational Health, College of Medicine, National Cheng Kung University, Tainan, Taiwan; 8 Department of Law, College of Social Science, National Cheng Kung University, Tainan, Taiwan; 9 Department of Environmental Health, Faculty of Public Health, University of Airlangga, Surabaya, Indonesia; 10 Department of Public Health, College of Public Health, China Medical University, Taichung, Taiwan; 11 Department of Healthcare Administration, College of Medical and Health Science, Asia University, Taichung, Taiwan

**Keywords:** Ambient air pollution, environmental health literacy, Cross-sectional studies, Community health, Linear regression model

## Abstract

**Objective:**

To investigate the level of and covariates associated with ambient air pollution health literacy (AAPHL) among adult residents of Taiwan.

**Methods:**

With a cross-sectional study design, we conducted telephone interviews using a Chinese version AAPHL scale, which consisted of 24 items assessing 12 subdomains of AAPHL formed by 4 information processing competence matrices (i.e., *access*, *understand*, *appraise*, and *apply*) and 3 health contexts (i.e., *healthcare*, *disease prevention*, and *health promotion*). The AAPHL was with the lowest and highest score at 1 to 4, respectively. Between September and November 2020, a sample of 1017 and 280 adults was successfully interviewed via home phones and mobile phones, respectively. We employed multiple linear regression models to identify covariates significantly associated with overall and 4 matric-specific AAPHL scores.

**Results:**

The mean and standard deviation (±SD) of overall AAPHL score was considered as moderate at 2.90 (±0.56), with the highest and lowest metric-specific score for “*apply*” (3.07 ± 0.59) and “*appraise*” (2.75 ± 0.66). Lower education was significantly associated with a lower overall score; and living with children < 12 years and single were both significantly associated with higher overall scores. We also noted a significant geographic variation in overall score in which people living in the east/remote islands had highest scores.

**Conclusions:**

People in Taiwan had only moderate level of AAPHL; and covariates including education, living arrangement, marital status, and area of living were significantly associated with AAPHL. These covariates should be considered in future educational interventions aiming to improve the AAPHL in the community.

**Supplementary Information:**

The online version contains supplementary material available at 10.1186/s12889-021-11658-z.

## Introduction

Ambient air pollution is a major environmental health problem affecting everyone in low-, middle-, and high-income countries; and representing a considerable threat to health worldwide [1]. According to the definition form WHO, ambient air pollution is a broader term used to describe air pollution in outdoor environments, while urban outdoor air pollution is a more specific term referring to the ambient air pollution experienced by populations living in urban areas, typically in or around cities [1, 2]. According to the 2015 Global Burden of Disease Study [3], exposure to ambient fine particulate matter PM_2.5_ is the fifth leading cause of death worldwide, accounting for 4.2 million deaths and 103.1 million disability-adjusted life-years in 2015 globally. Epidemiological studies reported links between air pollution and certain diseases of public health importance such as cardiovascular diseases, cancers, and respiratory diseases [4]. Recent studies also revealed potential influence of air pollution on psychiatric disorders [5, 6]. Taiwan is no exception [7]. Estimated in 2014, PM_2.5_ accounted for 6,282 deaths from ischemic heart disease, stroke, lung cancer, and chronic obstructive pulmonary disease, representing a population attributable mortality fraction of 18.6% associated with the four disease causes [7].

Ambient air pollution is a function of complex systems, and solutions to the problem also require multilevel intervention [8]; and education of people involved in the air pollution control strategies, including scientists, negotiators, decision makers and the public, to raise the environmental awareness is essential for reducing the air pollution [9]. Previous studies have demonstrated that environmental health education interventions at formal education or in the community could significantly have knowledge gains related to environmental health, individual behavior changes, and collective action for community change [10, 11]. Effective educational interventions were found to increase prevalence and effects of so-called avoidance behaviors in lowering the adverse effects of air pollution on health [12]. Such activities include purchasing preventive pharmaceuticals and reducing time spent in polluted environments [13].

The knowledge of environmental health and the behavior of environmental protection, which is so called environmental health literacy (EHL), with a root of both health literacy [14] and risk communication [10, 11], is an emerging area of study that incorporates content and strategies from environmental, health, and social sciences to promote understanding of the ways environmental contaminants affect health [15], which can be used as a tool for evaluating the effectiveness of environmental education. The earlier conceptual model of EHL was adapted from Bloom (1956), representing the stepwise progression of six distinct educational stages (i.e, create, evaluate, analyze, apply, understand, and recognize) to approach the development of targeted interventions for different levels of EHL [16]. The EHL on ambient air pollution can be enhanced by empowering individuals and communities to use appropriate communication for controlling air pollution exposures. However, to the best of our knowledge, previous air pollution assessments were focused on the air pollutants concentration or air quality monitoring [17, 18], and our study is the first one that assessed the level of ambient air pollution health literacy (AAPHL) in the general population. In fact, there is still a lack of easy and self-administrated checklist items like the one that we used in the current study for assessing the level of knowledge, competence, and motivation in dealing with ambient air pollution and human health. AAPHL which is defined as individual’s competencies to access, understand, appraise, and apply the ambient air pollution health information to make judgments and decisions concerning healthcare, disease prevention, and health promotion contexts to maintain or improve their quality of life and to protect the environment in urban and non-urban community [19]. The questionnaire was developed based on three rounds of consensus meetings including 5 experts of public health, environmental science, medical physicians, and air pollutant researchers in order to reflect the synergized subjective opinions of AAPHL. The content of the AAPHL scale included questions related to both urban and non-urban ambient air pollutant categories, air quality detection, pollutant sources, control strategies, law regulations, application of health-related information according to the above health literacy subdomains. Therefore, we developed the AAPHL questionnaire on the basis of an existing conceptual framework of health literacy proposed by the European Health Literacy Consortium, which composed of 12 subdomains of health literacy formed by 4 information processing competencies of individuals (ie, accessing, understanding, appraising, and applying) and 3 health contexts (ie, healthcare, disease prevention, and health promotion) [19]. Despite that, only very few studies have been conducted to address the issue of EHL on ambient air pollution and health threats [20, 21], and no survey data on ambient air pollution health literacy available at a population-based level. We therefore conducted this national population-based survey on level of AAPHL in adult residents of Taiwan.

## Methods

The study was ethnically approved by the National Chung Kung University Governance Framework for Human Research Ethics (No. 109–385).

### Study design and participants

This was a population-based cross-sectional study design. The target population was all Taiwanese residents aged 20 years and older. There are 22 cities/counties in Taiwan. By the end of 2019, a total of 19,338,629 adult (> = 20 years) residents including 9,486,379 men and 9,852,250 women were registered in the Household Registration [22]. These adults were inhabitants of 6,956,341 households all over the country. Seven covariates which are potential predictors (i.e., gender, age, education, and occupation as the personal determinants; living arrangement and marital status for situational determinants; living area as a socio-environmental determinants) for AAPHL scores were tested in this study on the basis of the integrated model of health literacy proposed by the European Health Literacy Survey Consortium [19].

The sample size required for this survey was calculated based on the multiple linear regression that identified factors significantly associated with AAPHL. Given that there were 7 potential covariates assessed for their associations with AAPHL, a sample size of 107 may achieve 90% power to detect a partial ρ^2^ of at least 0 (null hypothesis) attributed to 7 independent variable(s) when the significance level (alpha) is 0.050 and the actual value of ρ^2^ is 0.1 (alternative hypothesis). The corresponding sample size for a smaller actual value of ρ^2^ = 0.05 and ρ^2^ = 0.01 was 212 and 1,017, respectively (NCSS, LLC, Utah USA). Because the 7 covariates included in the models were potentially associated with health literacy (see the *Covariate* section below), we believe that they may have at least a partial ρ^2^ of 0.01, and as such a minimum of 1017 participants is needed for this study. The main purpose of cell phone survey was to increase the representativeness of those who do not have home phones. The number of 280 cell phone interviews was arbitrarily determined based on the availability of time and funding.

### Instrument and measurements of AAPHL

The Chinese version AAPHL scale was developed on the basis of The European Health Literacy Survey Questionnaire (HLS-EU-Q) [23]. The AAPHL was designed to be an integrated model of EHL, which comprised 24 items assessing 12 subdomains of EHL formed by 4 information processing competencies of individuals (i.e., accessing/obtaining information, understanding information, appraising/processing information, and applying/using information) and 3 health contexts (i.e., healthcare, disease prevention, and health promotion) to maintain or improve their quality of life and protect the environment in the community. The questionnaire was developed based on three rounds of consensus meetings including 5 experts of public health, environmental science, medical physicians, and air pollutant researchers in order to reflect the synergized subjective opinions of AAPHL. The content of the AAPHL scale included questions related to both urban and non-urban outdoor air pollutant categories, air quality detection, pollutant sources, control strategies, law regulations, application of health-related information according to the above health literacy subdomains. The HLS-EU-Q is highly recommended because it is founded on a testable conceptual framework, captures multiple conceptual domains of health literacy, and covers a diverse range of health contexts [24, 25]. Contents of the Chinese version AAPHL scale were displayed in Supplementary Table 1.

Two environmental epidemiologists and one environmental health scientist who have expertise on air pollution and human health were asked to perform content validity that assesses whether our AAPHL scale is representative of all aspects of the construct, namely the three health contexts: (1) healthcare; (2) disease prevention; (3) health promotion, and each context explored four health information processing competences: accessing/obtaining information; understanding information; processing/appraising information and applying/using information. The 4-point Liker’s scale was used to indicate the level of appropriateness for 3 categories in “*relevance*”, “*importance*”, and “*unambiguity*”, respectively of the AAPHL scale. The mean score for “*relevance*”, “*importance*”, and “*unambiguity*” of the scale was 3.90, 3.97, and 3.91, respectively; and the corresponding figures of Content Validity Index (CVI) were 0.97, 0.99, and 0.94, which were calculated from the method proposed by Aiken [26].

Apart from the content validity, we also examined the construct validity to evaluate whether the Chinese version AAPHL scale really represents the concept (i.e., construct) we are interested in measuring. Information of construct validity is central to establishing the overall validity of a method [27]. Based on the data of our study, we performed confirmatory factor analysis (CFA) to assess the construct validity. We performed a first-order CFA to verify the 12-subdomain factor structure of the Chinese version AAPHL scale. The first-order model was considered valid if the CFA demonstrated acceptable fit between the overall model and data on the basis of the following absolute and relative fit indices: (1) the χ^2^ test: it indicates the difference between observed and expected covariance matrices. Values closer to zero indicate a better fit; (2) the root mean squared error of approximation (RMSEA) of 0.08 or less [28]; (3) the standardized root mean square residual (SRMR) of 0.08 or less [29]; (4) the adjusted goodness of fit index (AGFI) of 0.80 or over [30]; and (5) both the normed fit index (NFI) and non-normed fit index (NNFI) of 0.90 or greater [29].

Although the χ^2^ test indicates a significant difference (*p* < 0.001) between observed and expected covariance matrices in our sample, the other fit indices tended to support the factorial validity of the 12-subdomain factor structure of the Chinese version AAPHL: RMSEA = 0.067; SRMR = 0.039; AGFI = 0.864; and NFI/NNFI = 0.9138/0.9021. It was thus recommended that the 12 subdomain scores be summed up to represent overall AAPHL. In addition, the factor loading for the 24 items of AAPHL ranged from 0.609 to 0.881, also suggesting an acceptable level [31].

The reliability of Chinese version AAPHL scale was determined by two internal consistence indicators. Based on our study sample, the Cronbach’s alpha was calculated at 0.934. Moreover, the composite reliability (i.e., construct reliability), a measure of internal consistency in scale items, much like Cronbach’s alpha [32] and can be thought of as being equal to the total amount of true score variance relative to the total scale score variance [33] showed that the composite reliability coefficient for the “access”, “understand”, “appraise”, and “apply” matrices was 0.852, 0.839, 0.845, and 0.798, respectively, which were all greater than an acceptable reliability level of 0.60 [32].

The possible responses and their scores for the AAPHL scale were as follows: very difficult = 1, fairly difficult = 2, fairly easy = 3, very easy = 4, and a “5” was indicated when participants did not answer or did not have a definite answer, coded as a missing value. The overall AAPHL score was calculated as the mean of all items applicable, scored from 1 to 4. Higher scores indicate better AAPHL. In addition to overall AAPHL score, we also calculated matric-specific score to indicate the information processing competencies of individuals, namely accessing, understanding, appraising, and applying matrices. Contents of the 24-item Chinese version AAPHL scale were displayed in Supplementary Table 1.

### Covariates

We collected the following covariates also via telephone interview to assess their associations with AAPHL level. The covariates included gender, age (20–34, 35–44, 45–54, 55–64, and > =65 years), education (Junior high school and lower, high school, college, and graduate studies), current occupation, living arrangement (living alone, living with children < 12 years, living with older [> = 12 years] students, or living with elderly [> = 65 years] people), marital status (single, married, others), and geographic area of living (north, central, south, and east/remote islands).

The currently held job was classified into one of 10 occupational categories: legislators, government administrators, business executives, and managers; professionals; technicians and associate professionals; clerks; service workers and shop and market sales workers; technology professionals; construction workers, agricultural, animal husbandry, forestry, and fishing workers; transportation and communication workers; teachers, athletes, and art performers; healthcare and social workers; legislators, government administrators, business executives and managers; and others (including housekeeper, retirees, and students). The occupational classification was based on the Standard Occupational Classification System (SOCS) of Taiwan for which interrater reliability has been shown to be good [34, 35].

Studies have shown that a number of factors may influence an individual’s health literacy, including living in poverty, education, race/ethnicity, age, and disability [36, 37]. In addition to socioeconomic status and co-morbidity, a recent study by Cho [38] indicated an association between work environment and level of health literacy. Because the above-mentioned covariates were reported to be associated with heath literacy, rather than with EHL or specifically with AAPHL, we examined in this study whether these sociodemographic and work characteristics also influence the AAPHL level.

### Data collection

The sampling method used in this study comprises two steps:. First, based on a predetermined total number of 1017 participants to be collected, we used the probability proportional to size (PPS) technique to determine the number of home phone to be called for each city/county according to the city/county specific population size [39, 40]. The PPS resulted in the number of call to be made for each city/county ranging from 37 to 385. Second, once the sample size was determined for each city/county we further employed a quota sampling by setting the age-specific sample size needed for each of the 7 age-specific populations (i.e., 20–29, 30–39, … ..80+) bases on the underlying age distributions of that particular city/county. The area codes and the first four digits of home phone numbers are unique for households in each city/county. The phone interviews continued until the predetermined sample size in each specific age group of specific city/county is met.

Considering an increasing number of residents in Taiwan only subscribed to mobile phones and some residents are usually not available during the period of making the home phone call, we additionally selected a supplemental sample via mobile phone. The mobile phone number is not city/county specific; therefore, the sample was selected from all mobile phone numbers of the entire country.

We employed the Computer-Assisted-Telephone Interview (CATI) to perform the interview. To achieve the predetermined age-specific sample size of sample for each city/county, we continuously performed the random digital dialing (RDD) procedure until the predetermined age-specific sample size was achieved. For each home phone called, only one eligible person best available in that household was invited. The CATI procedure reached a total of 4084 eligible adults via home phones, and 1017 adults successfully completed the home phone interviews, with a response rate of 24.90%. Reasons for unsuccessful home phone interviews were mainly due to the interviewees felt that the interview consumed more time than he/she expected and decided not to continue (19.9%), or the interviewees declined to be interviewed before the interview began (80.1%). The response rate (280/835 or 33.53%) was somewhat higher for the mobile phone interview as compared to the home phone.

The interview took around 15–20 min. The CATI was performed between September and November 2020 by five interviewers standardized to conduct telephone interviews. All phone calls were made between 5:00 PM and 10:00 PM to maximize the chance of reaching eligible subjects and to increase the likelihood of acceptance to be interviewed. Once the phone call reached a home phone/mobile phone, the people who answered the call were asked to determine his/her age eligibility. If the person who answered the call was eligible, he/she was invited. If not eligible or declined the invitation, any other eligible subject next him/her and available was invited.

Table 1 shows that the mobile phone interviewees tended to be female dominance, younger, more educated, living alone, single, and living in the north. Despite significant differences in socio-demographic characteristics between home phone and mobile phone interviewees, there were only slight differences in overall AAPHL and matric-specific scores, we therefore combined the home phone and mobile phone samples in the subsequent analyses to increase the representativeness of our study sample (Supplementary Table 2).

**Table 1:** Characteristics of study participants

Characteristics	Source of information				
	Home phones (*n* = 1017)		Mobile phones (*n* = 280)		*p* value
	*n*	%	*n*	%	
Gender					0.004
Men	473	46.5	103	36.8	
Women	544	53.5	177	63.2	
Age (years)					< 0.001
20–34	228	22.4	79	28.2	
35–44	180	17.7	65	23.2	
45–54	222	21.8	74	26.4	
55–64	257	25.3	53	18.9	
> =65	130	12.8	9	3.2	
Education					< 0.001
Junior high school and lower	118	11.6	7	2.5	
High school	306	30.1	67	23.9	
College	516	50.7	181	64.6	
Graduate studies	77	7.6	25	8.9	
Occupation					< 0.001
Clerks	52	5.2	30	10.9	
Service workers and shop and market sales workers	214	21.1	50	18.1	
Technology professionals	53	5.3	18	6.6	
Construction workers	51	5.1	14	5.1	
Agriculture, animal husbandry, forestry, and fishing workers	38	3.8	15	5.5	
Transportation and communication workers	59	5.9	30	10.9	
Teachers, athletes, and art performers	103	10.1	43	15.6	
Healthcare and social workers	27	2.7	12	4.4	
Legislators, government administrators, business executives and managers	37	3.7	14	5.1	
Others (including housekeeper, retirees, and students)	374	37.1	49	17.8	
Living arrangement					< 0.001
Living alone	93	9.1	54	19.3	
Living with someone	924	90.9	226	80.7	
Children < 12 years	225	22.1	44	15.7	
Someone who is a student and aged > = 12 years	407	40.0	102	36.4	
Elderly people aged > = 65	420	41.3	90	32.1	
Marital status					0.006
Single	268	26.4	93	33.2	
Married	698	68.6	145	51.8	
Others	51	5.0	42	15.0	
Area of living					< 0.001
North	457	44.9	179	63.9	
Central	240	23.6	45	16.1	
South	257	25.3	46	16.4	
East and remote islands	63	6.2	10	3.6	

### Statistical analysis

We first presented characteristics of the study participants with numbers and percentages. The AAPHL score was presented with mean and standard deviation (SD), and comparison of the differences among the 4 matric-specific AAPHL scores was made with repeated measurement analysis of variance, which considers the inter-correlation of the matric-specific scores made by the same interviewee. Multiple linear regression analysis was performed to investigate the covariates significantly associated with overall and matric-specific AAPHL score separately. We checked the assumptions of linearity, normality, and homoscedasticity for linear regression model by examining the residual plots, and found no violation of the above assumptions.

All statistical analyses were performed using SAS statistical software (SAS System for Windows, Version 9.4, SAS Institute Inc., Cary, NC, USA). Results with two-sided *P* values less than .05 were considered statistically significant.

## Results

**Fig. 1:**
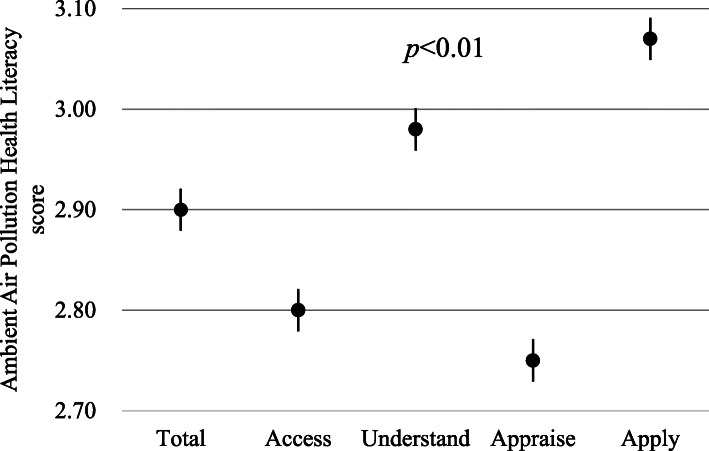
Overall and matric-specific scores of the *Ambient Air Pollution Health Literacy* (AAPHL)

Figure 1 shows the mean and SD of overall and 4 matric-specific AAPHL scores. Supplementary Table 2 further shows distributions of overall and metric-specific scores of AAPHL according to phone type. The mean and SD of overall AAPHL score was considered as moderate at 2.90 (0.56), with significant variation in metric-specific AAPHL. The highest and lowest metric-specific mean ± SD score was noted for “apply” (3.07 ± 0.59) and “appraise” (2.75 ± 0.66), respectively. Each metric-specific AAPHL score showed a high correlation with overall AAPHL score, with a Spearman’s correlation coefficient ranging from 0.83 (“apply” and overall) to 0.90 (“understand” and overall). However, the inter-correlation between the 4 metric-specific AAPHL scores was only moderate at 0.61 (“access” and “apply”) to 0.74 (“access” and “understand”) (Table 2).

**Table 2:** Inter-correlation among total and matric-specific scores of ambient air pollution health literacy

Matrices	Access	Understand	Appraise	Apply	Total
Access	1.00 ^a^	0.74	0.63	0.61	0.87
Understand		1.00	0.70	0.68	0.90
Appraise			1.00	0.62	0.85
Apply				1.00	0.83
Total					1.00

^a^ Indicated by Spearman correlation coefficient

Table 3 shows the results of multiple linear regression analysis. Covariates significantly associated with overall AAPHL score included education, living arrangement, marital status, and area of living. Compared to those with college education, the participants with education levels of high school (adjusted *β* = − 0.09) and junior high school and lower (adjusted *β* = − 0.15) had significantly lower overall scores. The participants living with children < 12 years (adjusted *β* = 0.15, with reference to those living alone) and those who were single (adjusted *β* = 0.10, with reference to those married) had significantly higher AAPHL scores. Compared to living in the north, living in central (adjusted *β* = 0.09) and east/remote islands (adjusted *β* = 0.25) were both associated with significantly higher overall AAPHL scores.

**Table 3:** Multiple linear regression models of ambient air pollution health literacy score in relation to socio-demographic characteristics (*n* = 1297)

Characteristics	Mean ± SD	Adjusted *β*	*p*-value
Gender			
Men	2.89 ± 0.59	Ref.	Ref.
Women	2.91 ± 0.53	0.02	0.46
Age (years)			
20–34	2.96 ± 0.57	Ref.	Ref.
35–44	2.89 ± 0.57	−0.004	0.94
45–54	2.88 ± 0.53	0.01	0.86
55–64	2.91 ± 0.56	0.06	0.25
> =65	2.87 ± 0.54	0.02	0.81
Education			
Junior high school and lower	2.81 ± 0.56	−0.15	0.01
High school	2.85 ± 0.58	−0.09	0.02
College	2.93 ± 0.54	Ref.	Ref.
Graduate studies	3.02 ± 0.52	0.08	0.20
Occupation			
Clerks	2.85 ± 0.53	−0.05	0.46
Service workers and shop and market sales workers	2.86 ± 0.60	Ref.	Ref.
Technology professionals	3.01 ± 0.53	0.11	0.14
Construction workers	2.87 ± 0.71	−0.002	0.98
Agriculture, animal husbandry, forestry, and fishing workers	2.93 ± 0.42	0.03	0.69
Transportation and communication workers	2.80 ± 0.47	−0.07	0.33
Teachers, athletes, and art performers	2.96 ± 0.53	0.06	0.35
Healthcare and social workers	2.86 ± 0.63	−0.04	0.68
Legislators, government administrators, business executives and managers	2.95 ± 0.49	0.01	0.88
Others (including housekeeper, retirees, and students)	2.92 ± 0.55	0.07	0.16
Living arrangement			
Living alone	2.92 ± 0.65	Ref.	Ref.
Living with	2.90 ± 0.54	0.01	0.84
Children < 12 years	3.00 ± 0.48	0.15	0.04
Someone who is a student and aged > = 12 years	2.88 ± 0.56	0.03	0.64
Elderly people aged > = 65	2.88 ± 0.53	−0.03	0.56
Marital status			
Single	2.98 ± 0.54	0.10	0.02
Married	2.88 ± 0.56	Ref.	Ref.
Others	2.86 ± 0.57	0.01	0.84
Area of living			
North	2.86 ± 0.58	Ref.	Ref.
Central	2.94 ± 0.51	0.09	0.02
South	2.90 ± 0.55	0.05	0.23
East and remote islands	3.13 ± 0.47	0.25	< 0.01

Supplementary Tables 3 to 6 showed the covariates in association with the 4 metric-specific AAPHL scores individually. Lower education was significantly associated with lower scores of all matrices except “appraise” metric. In addition, adults living with children < 12 years had significantly higher scores in both “appraisal” and “apply” metrices; and single was significantly associated with higher scores of all matrices except “appraisal”. A significant geographic variation in AAPHL score was observed for all matrices, with participants from east/remote islands had consistently higher scores. Some occupations were found to be sporadically associated with certain metric-specific AAPHL scores.

## Discussion

To the best of our knowledge, this is the first study developing the measurement tool of EHL in ambient air pollution, exploring the level of AAPHL nationally, and identifying covariates significantly associated with AAPHL score. For the AAPHL assessment, we employed the conceptual framework proposed by European Health Literacy Consortium. The comprehensive questionnaire of HLS-EU-Q47 not only assesses the concept of HL in terms of 3 health contexts (health care, disease prevention, and health promotion) and four competencies regarding health information (“access”, “understanding”, “appraisal”, and “apply”), but also encompasses the antecedents and consequences of HL [19]. People with chronic diseases (particularly cardiorespiratory illnesses) [41, 42], little social support, and poor access to medical services are most at risk from air pollution [43]. Therefore, exploring the level of ambient air pollution health-related skills and identifying key covariates associated with these skills may help guide policies to improve the understanding of the link between air pollution exposures and health.

EHL is a natural outgrowth concept incorporating health literacy, public health, environmental health science, and environmental literacy to develop the wide range of skills and competencies linking between environmental exposures and health [10]. Therefore, it is a must to assess the ability of population to seek out, comprehend, evaluate, and use the health information regarding to air pollution, as well as to make informed choices, reduce health risks, improve quality of life and protect the environment [11]. Both overall and matric-specific AAPHL scores in our survey revealed a sufficient (2.7–3.1 out of 4) level of health literacy which is similar as our previous study [44]. Our study showed the self-reported difficulty of AAPHL competency from the easiest to the most difficult is “apply”, “understand”, “access”, and “appraisal”. This finding is similar to the result from the previous survey conducted in eight Europeans countries proposing that tasks relating to “appreciation” or “accessing” of information are perceived as more difficult than “understanding” or “applying” information [45]. In addition, our study revealed that all four information processing competence measures have high correlation with the overall score of AAPHL. Among the four competences of “access”, “understand”, “appraise”, and “apply” the ambient air pollution health information, the matric of “understand” correlates most to the total AAPHL while the matric of “apply” correlates least with the other three competence matrices. This implied that our current self-reported AAPHL questionnaire measures more air pollution health literacy related domains of knowledge level than skill or behavior concepts. This barrier in measuring health literacy had been noticed by a Canadian study which emphasized the “two-sided” nature of health literacy, both the knowledge and skill that improve the ability of people to act on information in order to live healthier lives [46].

Recently, a model integrating public health and health care views of general health literacy has been proposed by the European Health Literacy Survey Consortium, which encompasses the 12 matrices concept of health literacy, determinants, and consequences of health literacy [19]. Among the determinants of health literacy proposed by EU, distinction factors include personal, situational, and socio-environmental determinants [19]. This is consistent with our study results indicating that the determinants of AAPHL include education attainment (personal determinants), living arrangement (situational determinants), marital status (situational determinants), and area of living (socio-environmental determinants). As for the personal determinants of the AAPHL score, our study disclosed that lower educational level is a significant influencing factor of AAPHL score, which is similar to those from a previous systematic review for heart failure population [47] and cross-sectional survey of community dwelling older adults [48].

Among the situational determinants, our results demonstrated both living with children below 12 years old and single status were factors significantly associated with higher levels of AAPHL. Previous epidemiological studies strongly suggested that air pollution damages vulnerable children’s health and its toxic effects not only occurring at the air-tissue interface of the lung but also affecting on other organs [49]. The government of Taiwan currently implements a health policy in which kindergartens and elementary school should raise colored flag (i.e., which green, yellow, orange, red, purple, and brown respectively) [50] according to the air quality index (AQI) announced by the Taiwan Environmental Protection Administration [51]. This may explain our study result showed a better AAPHL score among participants living with children below 12 years old. As for the other situational determinants of health literacy, our results revealed that participant who were single had better AAPHL scores as compared to those married ones. Individuals living alone were likely to have higher exposures to ambient air pollutants monitoring as they have to go outside often for work or shopping, which in turns may raise their awareness of ambient air pollution [52]. One previous study proposed that single breast cancer survivors would have better generic health literacy and involvement of medical decisions [52, 53].

Since AAPHL is a measure related to EHL, certain socio-environmental determinants such as living environment is also a critical factor affecting the awareness of ambient air pollution and health. Our study showed that people living in east and remote islands has higher AAPHL scores than living the other areas. A previous systematic review has mentioned that an association between air quality index and pollution risk perception is existed through the influence of behavior, experience, socioeconomic factors, and information/communication [54]. This might explain our study result because most of the time in Taiwan. According to the Air Quality Monitoring Network of Taiwan Environmental Protection Administration, the air quality is better in the east and remote islands. We speculated the observation that where people from the areas with better air quality tended to have higher awareness of air pollution and health could be related to the frequent self-rescue campaign for ecological conservation and environmental protection against the build-up of petrochemical plants in the neighborhood initiated by local indigenous residents, which might also lead to a success of certain local ambient air pollution control programs and activities.

This study has several limitations. First, due to the cross-sectional study design, causal relationships cannot be established. Second, we cannot rule out potential selection bias because the telephone interview survey would exclude people without home phones or mobile phones. In addition, people who were willing to respond to interview might be different from those who were not, which might have entails at least in some extent certain degrees of selection bias. Although the home phone participants were sampled using PPS method, and a cell phone sample was collected to increase the representativeness of the study sample, low response rates for home phone (24.90%) and cell phone (33.5%) surveys might compromise the truthfulness about some sensitive issues, such as education and occupation. Third, the AAPHL were self-reported in our study, which is subject to information bias. However, some studies have found no differences between self-reported and performance-based health literacy measures [55]. Last, although we have included some socioeconomic status (e.g., occupation, education, living status) as covariates which might influence the level of AAPHL, we did not collect information of medical conditions so that we were unable to further identify certain subgroups (e.g., existing chronic diseases, social support, medical access, etc.) potentially vulnerable to lower level of AAPHL. Additionally, owing to incomplete adjustment for the aforementioned known risk factors for health literacy of ambient air pollution, a potential for residual confounding cannot be entirely excluded. The generalisability of the results may also be limited as our sample was comprised of a larger portion of participants with higher socioeconomic status (i.e., 58% graduated from college or above) than the general population.

## Conclusion

Adult residents in Taiwan had only moderate level of AAPHL; and education, living arrangement, marital status, and area of living were significantly associated with AAPHL. Because ambient air pollution is local and air quality varies seasonally and throughout the day, our study results may provide evidence-based health polity for researchers to consider tailored educational intervention programs with the consideration of personal, situational, and socio-environmental determinants to improve the AAPHL in the community; as well as for practitioners to provide an effective risk communication about air quality with local needs and real-time information across each community.

## Supplementary Information

**Additional file 1.**

## Data Availability

The datasets used and/or analyzed during the current study are available from the corresponding author on reasonable request.

## Abbreviations

AAPHL: Ambient Air Pollution Health Literacy
EHL: Environmental Health Literacy
HLS-EU-Q: European Health Literacy Survey Questionnaire
CVI: Content Validity Index
CFA: Confirmatory Factor Analysis
RMSEA: Root Mean Squared Error of Approximation
SRMR: Standardized Root Mean Square Residual
AGFI: Adjusted Goodness of Fit Index
NFI: Normed Fit Index
NNFI: Non-Normed Fit Index
PPS: Probability Proportional to Size
CATI: Computer-Assisted-Telephone Interview
RDD: Random Digital Dialing
SD: Standard Deviation
AQI: Air Quality Index
