# Supplementary material for: A national survey of ambient air pollution health literacy among adult residents of Taiwan
Source: BMC Public Health. 2021 Aug 31;21:1604. doi: 10.1186/s12889-021-11658-z (PMC8406719; doi:10.1186/s12889-021-11658-z)
Supplement: Supplementary file 1 — Additional file 1. [file 12889_2021_11658_MOESM1_ESM.docx]

**Supplementary Material Legends**

Supplementary Table 1. List of the items of *Ambient air pollution Health Literacy* (AAPHL) scale

Supplementary Table 2. Comparisons of overall and matric-specific ambient air pollution health literacy between home-phone sample and mobile phone sample

Supplementary Table 3. Multiple linear regression models of ambient air pollution health literacy score (***access***) in relation to socio-demographic characteristics (*n*=1,297)

Supplementary Table 4. Multiple linear regression models of ambient air pollution health literacy score (***understand***) in relation to socio-demographic characteristics (*n*=1,297)

Supplementary Table 5. Multiple linear regression models of ambient air pollution health literacy score (***appraise***) in relation to socio-demographic characteristics (*n*=1,297)

Supplementary Table 6. Multiple linear regression models of ambient air pollution health literacy score (***apply***) in relation to socio-demographic characteristics (*n*=1,297)

Supplementary Table 1. List of the items of *Ambient air pollution Health Literacy* (AAPHL) scale

|  | | Level of difficulty | | | | |
| --- | --- | --- | --- | --- | --- | --- |
| For the sake of your own health, we would like to understand your ability to recognize **the influences of ambient air pollution** **on health**. Please evaluate the difficulty of accessing, *understanding*, *appraising* and *applying* of air pollution information based on the three aspects of health promotion, disease prevention and health care, and check the answers.  Note: If you know the title description, but have never touched it or have never done it, please check "not experienced"; if you are not sure about the content of the title, please check "Don't know". | | 1 | 2 | 3 | 4 | 5 |
| 1 | When you want to know the influence of air pollution on health, is it easy for you to **access** information? |  |  |  |  |  |
| 2 | When you want to know the status of air quality, is it easy for you to **access** information? |  |  |  |  |  |
| 3 | Is it easy for you to **understand** the air quality index provided on the Environmental Protection Agency (EPA) website, weather reports of Central Weather Bureau or the information released from government media? |  |  |  |  |  |
| 4 | Is it easy for you to **understand** influences of poor air quality on the health? |  |  |  |  |  |
| 5 | Is it easy for you to **evaluate** the air quality of your living environment (including the community and neighborhood you live in)? |  |  |  |  |  |
| 6 | Is it easy for you to **judge** that some social media’s suggestions for improving air quality are incorrect? |  |  |  |  |  |
| 7 | Is it easy for you to **engage** in suitable outdoor activities based on the air quality index provided by the EPA? |  |  |  |  |  |
| 8 | Is it easy for you to **adopt** the air pollution improvement suggestions put forward by the government? |  |  |  |  |  |
| 9 | Is it easy for you to **access** information about reducing the health hazards of air pollution? |  |  |  |  |  |
| 10 | Is it **easy** for you to find out what the government has done to reduce air pollution? |  |  |  |  |  |
| 11 | Can you **understand** which air pollutants are harmful to human health? Annotation: According to the "Enforcement Rules of the Air Pollution Prevention and Control Law", the "air pollutants" in the title include gaseous pollutants, particulate pollutants, derivative pollutants, harmful air pollutants and odor pollutants, such as: PM2.5, factories' black smoke, household oil fume, Dioxin after burning plastic products, etc. |  |  |  |  |  |
| 12 | Do you **understand** why the government monitors the level of ambient air pollution? |  |  |  |  |  |
| 13 | Can you **judge** whether the information provided by social media about diseases caused by air pollution is correct? |  |  |  |  |  |
| 14 | Can you **judge** whether the "methods to reduce the health hazards of air pollution" provided by social media are credible? |  |  |  |  |  |
| 15 | Do you know how you can **reduce** the chance of diseases caused by air pollution? (For example: when do you need to reduce outdoor activities or choose a suitable place) |  |  |  |  |  |
| 16 | Will you have your cars and motorcycles **inspected regularly** within the time limit set by the government to reduce air pollution and possible diseases caused by air pollution? |  |  |  |  |  |
| 17 | Can you **find** relevant government regulations regarding air pollution control? Annotation: The laws and regulations related to air pollution prevention and control include: <Air Pollution Prevention and Control Law>, <Implementation Rules of Air Pollution Prevention and Control Law>, <Emergency Prevention Measures for Severe Air Quality Deterioration>, and <County and City Governments (especially municipalities) related Autonomous regulations, such as: Kaohsiung City Environmental Maintenance and Management Autonomous Regulations (Chapter III Air Pollution Management)> etc. |  |  |  |  |  |
| 18 | Can you **access** related information on ways to improve the air quality in your surroundings? Annotation: Here are ways to make the surrounding air quality better, including: reduce driving and use public transportation, plant more green plants, etc. |  |  |  |  |  |
| 19 | Do you **understand** how the government provides subsidies for the purchase of electric vehicles to reduce air pollution? |  |  |  |  |  |
| 20 | Can you **understand** the contents of Taiwan's air pollution control act? Annotation: <Air Pollution Prevention Measures> include the delineation of air pollution prevention and control areas at all levels, emergency prevention and control of severe air quality deterioration, subsidies for the purchase of electric locomotives, prohibition of the use of two-stroke locomotives, mandatory catering industry to install exhaust systems, and regular mobile pollution sources testing, delimitation of total volume control area, setting up air quality monitoring facilities, etc. |  |  |  |  |  |
| 21 | Can you **judge** the correctness of the content of the health promotion methods proposed by radio, television or online media that can reduce the impact of air pollution? Annotation: Take public tools, refrain from smoking in non-smoking places, and purchase electric cars and motorcycles in accordance with policies. |  |  |  |  |  |
| 22 | Can you **judge** whether the government's air pollution reduction subsidy policy is suitable for you? Annotation: The air pollution reduction subsidy policy is based on the policy put forward by the "Air Pollution Prevention Action Plan", such as the promotion of subsidies for electric cars and locomotives, replacement subsidies for energy-saving appliances, etc. |  |  |  |  |  |
| 23 | Do you usually **choose** non-smoking roads, non-smoking parks, or non-smoking places of business to protect your health? |  |  |  |  |  |
| 24 | Can you **make decisions** for your own health (such as wearing a mask) and reducing air pollution (such as reducing driving and changing to use public transportation)? |  |  |  |  |  |

1: Very difficult; 2: Fairly difficult; 3: Fairly easy; 4: Very easy; 5: Not known or no experience

Supplementary Table 2. Comparisons of overall and matric-specific ambient air pollution health literacy between home-phone sample and mobile phone sample

|  | Mean±SD | Min. | Q1 | Med. | Q3 | Max. |
| --- | --- | --- | --- | --- | --- | --- |
| Total |  |  |  |  |  |  |
| Home phone sample | 2.93±0.54 | 1 | 2.55 | 2.92 | 3.30 | 4 |
| Mobile phone sample | 2.82±0.60 | 1 | 2.44 | 2.81 | 3.22 | 4 |
| Total sample | 2.90±0.56 | 1 | 2.53 | 2.90 | 3.29 | 4 |
| Access |  |  |  |  |  |  |
| Home phone sample | 2.81±0.69 | 1 | 2.33 | 2.83 | 3.33 | 4 |
| Mobile phone sample | 2.74±0.70 | 1 | 2.23 | 2.71 | 3.17 | 4 |
| Total sample | 2.80±0.69 | 1 | 2.33 | 2.83 | 3.33 | 4 |
| Understand |  |  |  |  |  |  |
| Home phone sample | 3.01±0.61 | 1 | 2.67 | 3.00 | 3.50 | 4 |
| Mobile phone sample | 2.88±0.70 | 1 | 2.40 | 3.00 | 3.40 | 4 |
| Total sample | 2.98±0.63 | 1 | 2.60 | 3.00 | 3.50 | 4 |
| Appraise |  |  |  |  |  |  |
| Home phone sample | 2.77±0.65 | 1 | 2.33 | 2.80 | 3.17 | 4 |
| Mobile phone sample | 2.67±0.69 | 1 | 2.17 | 2.60 | 3.17 | 4 |
| Total sample | 2.75±0.66 | 1 | 2.33 | 2.75 | 3.17 | 4 |
| Apply |  |  |  |  |  |  |
| Home phone sample | 3.09±0.58 | 1 | 2.67 | 3.00 | 3.50 | 4 |
| Mobile phone sample | 2.98±0.62 | 1 | 2.60 | 3.00 | 3.50 | 4 |
| Total sample | 3.07±0.59 | 1 | 2.67 | 3.00 | 3.50 | 4 |

Min., minimum; Q1, the 25^th^ percentile; Med., median; Q3, the 75^th^ percentile; Max., maximum

Supplementary Table 3. Multiple linear regression models of ambient air pollution health literacy score (***access***) in relation to socio-demographic characteristics (*n*=1,297)

| Characteristics | Mean±SD | Adjusted *β* | *p*-value |
| --- | --- | --- | --- |
| Gender |  |  |  |
| Men | 2.76±0.74 | Ref | Ref |
| Women | 2.83±0.67 | 0.08 | 0.04 |
| Age (years) |  |  |  |
| 20-34 | 2.96±0.69 | Ref | Ref |
| 35-44 | 2.82±0.66 | -0.02 | 0.71 |
| 45-54 | 2.76±0.68 | -0.04 | 0.50 |
| 55-64 | 2.72±0.70 | -0.03 | 0.70 |
| >=65 | 2.62±0.74 | -0.09 | 0.29 |
| Education |  |  |  |
| Junior high school and lower | 2.42±0.70 | -0.45 | <0.01 |
| High school | 2.69±0.70 | -0.18 | <0.01 |
| College | 2.88±0.68 | Ref | Ref |
| Graduate studies | 3.00±0.59 | 0.12 | 0.11 |
| Occupation |  |  |  |
| Clerks | 2.82±0.64 | -0.04 | 0.62 |
| Service workers and shop and market sales workers | 2.75±0.72 | Ref | Ref |
| Technology professionals | 2.94±0.64 | 0.08 | 0.39 |
| Construction workers | 2.83±0.85 | 0.05 | 0.60 |
| Agriculture, animal husbandry, forestry, and fishing workers | 2.83±0.58 | 0.03 | 0.76 |
| Transportation and communication workers | 2.70±0.60 | -0.08 | 0.32 |
| Teachers, athletes, and art performers | 2.91±0.67 | 0.07 | 0.29 |
| Healthcare and social workers | 2.63±0.76 | -0.20 | 0.09 |
| Legislators, government administrators, business executives and managers | 2.88±0.59 | -0.02 | 0.88 |
| Others (including housekeeper, retirees, and students) | 2.76±0.71 | 0.06 | 0.32 |
| Living arrangement |  |  |  |
| Living alone | 2.86±0.73 | Ref | Ref |
| Living with someone | 2.79±0.69 | -0.03 | 0.64 |
| Children <12 years | 2.88±0.64 | 0.14 | 0.14 |
| Someone who is a student and aged >=12 years | 2.79±0.71 | 0.01 | 0.84 |
| Elderly people aged >=65 | 2.76±0.69 | -0.08 | 0.26 |
| Marital status |  |  |  |
| Single | 2.96±0.66 | 0.14 | 0.01 |
| Married | 2.74±0.70 | Ref | Ref |
| Others | 2.70±0.70 | -0.03 | 0.69 |
| Area of living |  |  |  |
| North | 2.76±0.70 | Ref | Ref |
| Central | 2.86±0.65 | 0.12 | 0.01 |
| South | 2.74±0.72 | -0.01 | 0.85 |
| East and remote islands | 3.07±0.64 | 0.32 | <0.01 |

Supplementary Table 4. Multiple linear regression models of ambient air pollution health literacy score (***understand***) in relation to socio-demographic characteristics (*n*=1,297)

| Characteristics | Mean±SD | Adjusted *β* | *p*-value |
| --- | --- | --- | --- |
| Gender |  |  |  |
| Men | 2.98±0.67 | Ref | Ref |
| Women | 2.99±0.59 | 0.02 | 0.51 |
| Age (years) |  |  |  |
| 20-34 | 3.04±0.65 | Ref | Ref |
| 35-44 | 2.98±0.65 | 0.001 | 0.99 |
| 45-54 | 2.98±0.59 | 0.05 | 0.43 |
| 55-64 | 2.96±0.63 | 0.07 | 0.24 |
| >=65 | 2.92±0.62 | 0.05 | 0.53 |
| Education |  |  |  |
| Junior high school and lower | 2.85±0.68 | -0.18 | 0.01 |
| High school | 2.91±0.66 | -0.11 | 0.01 |
| College | 3.02±0.61 | Ref | Ref |
| Graduate studies | 3.14±0.54 | 0.10 | 0.16 |
| Occupation |  |  |  |
| Clerks | 2.94±0.58 | -0.04 | 0.60 |
| Service workers and shop and market sales workers | 2.93±0.69 | Ref | Ref |
| Technology professionals | 3.19±0.61 | 0.21 | 0.01 |
| Construction workers | 2.94±0.77 | -0.002 | 0.98 |
| Agriculture, animal husbandry, forestry, and fishing workers | 2.96±0.50 | -0.002 | 0.98 |
| Transportation and communication workers | 2.93±0.55 | -0.004 | 0.96 |
| Teachers, athletes, and art performers | 3.10±0.60 | 0.11 | 0.08 |
| Healthcare and social workers | 2.87±0.69 | -0.11 | 0.31 |
| Legislators, government administrators, business executives and managers | 3.02±0.57 | -0.001 | 0.99 |
| Others (including housekeeper, retirees, and students) | 2.96±0.61 | 0.03 | 0.53 |
| Living arrangement |  |  |  |
| Living alone | 2.97±0.74 | Ref | Ref |
| Living with someone | 2.99±0.61 | 0.04 | 0.47 |
| Children <12 years | 3.11±0.54 | 0.17 | 0.06 |
| Someone who is a student and aged >=12 years | 2.96±0.65 | 0.07 | 0.34 |
| Elderly people aged >=65 | 2.97±0.59 | -0.05 | 0.94 |
| Marital status |  |  |  |
| Single | 3.06±0.61 | 0.10 | 0.04 |
| Married | 2.96±0.64 | Ref | Ref |
| Others | 2.90±0.59 | -0.04 | 0.61 |
| Area of living |  |  |  |
| North | 2.93±0.65 | Ref | Ref |
| Central | 3.02±0.60 | 0.12 | 0.01 |
| South | 3.02±0.62 | 0.11 | 0.01 |
| East and remote islands | 3.17±0.55 | 0.24 | <0.01 |

Supplementary Table 5. Multiple linear regression models of ambient air pollution health literacy score (***appraise***) in relation to socio-demographic characteristics (*n*=1,297)

| Characteristics | Mean±SD | Adjusted *β* | *p*-value |
| --- | --- | --- | --- |
| Gender |  |  |  |
| Men | 2.75±0.69 | Ref | Ref |
| Women | 2.75±0.64 | 0.01 | 0.76 |
| Age (years) |  |  |  |
| 20-34 | 2.77±0.69 | Ref | Ref |
| 35-44 | 2.70±0.65 | -0.04 | 0.54 |
| 45-54 | 2.71±0.66 | -0.003 | 0.96 |
| 55-64 | 2.78±0.66 | 0.09 | 0.17 |
| >=65 | 2.78±0.62 | 0.09 | 0.30 |
| Education |  |  |  |
| Junior high school and lower | 2.77±0.68 | -0.03 | 0.65 |
| High school | 2.74±0.67 | -0.01 | 0.80 |
| College | 2.74±0.65 | Ref | Ref |
| Graduate studies | 2.78±0.63 | 0.04 | 0.58 |
| Occupation |  |  |  |
| Clerks | 2.69±0.65 | -0.02 | 0.79 |
| Service workers and shop and market sales workers | 2.73±0.69 | Ref | Ref |
| Technology professionals | 2.83±0.64 | 0.12 | 0.20 |
| Construction workers | 2.75±0.83 | 0.03 | 0.72 |
| Agriculture, animal husbandry, forestry, and fishing workers | 2.80±0.56 | 0.06 | 0.55 |
| Transportation and communication workers | 2.66±0.51 | -0.05 | 0.57 |
| Teachers, athletes, and art performers | 2.76±0.66 | 0.02 | 0.77 |
| Healthcare and social workers | 2.66±0.66 | -0.09 | 0.45 |
| Legislators, government administrators, business executives and managers | 2.74±0.61 | -0.02 | 0.88 |
| Others (including housekeeper, retirees, and students) | 2.77±0.66 | 0.03 | 0.61 |
| Living arrangement |  |  |  |
| Living alone | 2.77±0.75 | Ref | Ref |
| Living with someone | 2.75±0.65 | -0.01 | 0.82 |
| Children <12 years | 2.83±0.63 | 0.10 | 0.33 |
| Someone who is a student and aged >=12 years | 2.72±0.68 | -0.03 | 0.71 |
| Elderly people aged >=65 | 2.73±0.65 | -0.05 | 0.50 |
| Marital status |  |  |  |
| Single | 2.78±0.68 | 0.06 | 0.22 |
| Married | 2.74±0.65 | Ref | Ref |
| Others | 2.70±0.65 | -0.01 | 0.90 |
| Area of living |  |  |  |
| North | 2.68±0.68 | Ref | Ref |
| Central | 2.81±0.63 | 0.15 | <0.01 |
| South | 2.79±0.63 | 0.13 | 0.01 |
| East and remote islands | 2.94±0.67 | 0.25 | <0.01 |

Supplementary Table 6. Multiple linear regression models of ambient air pollution health literacy score (***apply***) in relation to socio-demographic characteristics (*n*=1,297)

| Characteristics | Mean±SD | Adjusted *β* | *p*-value |
| --- | --- | --- | --- |
| Gender |  |  |  |
| Men | 3.07±0.63 | Ref | Ref |
| Women | 3.06±0.56 | -0.03 | 0.42 |
| Age (years) |  |  |  |
| 20-34 | 3.07±0.61 | Ref | Ref |
| 35-44 | 3.02±0.61 | 0.02 | 0.74 |
| 45-54 | 3.05±0.57 | 0.04 | 0.47 |
| 55-64 | 3.11±0.56 | 0.09 | 0.12 |
| >=65 | 3.10±0.60 | 0.03 | 0.71 |
| Education |  |  |  |
| Junior high school and lower | 3.08±0.62 | -0.06 | 0.29 |
| High school | 3.01±0.60 | -0.10 | 0.02 |
| College | 3.09±0.58 | Ref | Ref |
| Graduate studies | 3.13±0.56 | 0.04 | 0.55 |
| Occupation |  |  |  |
| Clerks | 2.94±0.57 | -0.09 | 0.21 |
| Service workers and shop and market sales workers | 3.00±0.61 | Ref | Ref |
| Technology professionals | 3.11±0.55 | 0.07 | 0.36 |
| Construction workers | 2.98±0.73 | -0.04 | 0.67 |
| Agriculture, animal husbandry, forestry, and fishing workers | 3.10±0.44 | 0.05 | 0.56 |
| Transportation and communication workers | 2.91±0.58 | -0.12 | 0.11 |
| Teachers, athletes, and art performers | 3.07±0.55 | 0.04 | 0.54 |
| Healthcare and social workers | 3.12±0.67 | 0.10 | 0.33 |
| Legislators, government administrators, business executives and managers | 3.14±0.51 | 0.07 | 0.46 |
| Others (including housekeeper, retirees, and students) | 3.14±0.59 | 0.14 | 0.01 |
| Living arrangement |  |  |  |
| Living alone | 3.05±0.70 | Ref | Ref |
| Living with someone | 3.07±0.57 | 0.05 | 0.40 |
| Children <12 years | 3.15±0.52 | 0.18 | 0.04 |
| Someone who is a student and aged >=12 years | 3.07±0.57 | 0.09 | 0.18 |
| Elderly people aged >=65 | 3.05±0.56 | 0.03 | 0.66 |
| Marital status |  |  |  |
| Single | 3.10±0.58 | 0.09 | 0.05 |
| Married | 3.05±0.60 | Ref | Ref |
| Others | 3.10±0.58 | 0.10 | 0.14 |
| Area of living |  |  |  |
| North | 3.06±0.61 | Ref | Ref |
| Central | 3.05±0.53 | 0.01 | 0.83 |
| South | 3.04±0.60 | -0.01 | 0.85 |
| East and remote islands | 3.31±0.51 | 0.23 | <0.01 |
